# Associations between Long-Term Dietary Coenzyme Q10 Intake and New-Onset Hypertension in Adults: Insights from a Nationwide Prospective Cohort Study

**DOI:** 10.3390/nu16152478

**Published:** 2024-07-31

**Authors:** Dan Zhao, Zezhong Tian, Huiying Kuang, Yixuan Xu, Yiqi Zheng, Zepei Zhong, Lihan Liang, Yan Yang

**Affiliations:** 1 School of Public Health (Shenzhen), Shenzhen Campus of Sun Yat-sen University, Sun Yat-sen University, Shenzhen 518107, Chinalianglh6@mail2.sysu.edu.cn (L.L.); 2 Guangdong Engineering Technology Center of Nutrition Transformation, Sun Yat-sen University, Shenzhen 518107, China; 3 Guangdong Provincial Key Laboratory of Food, Nutrition and Health, Sun Yat-sen University, Guangzhou 510080, China; 4 Zhongshan School of Medicine, Sun Yat-sen University, Guangzhou 510080, China

**Keywords:** CoQ10, blood pressure, non-linear relationship, primary prevention

## Abstract

Coenzyme Q10 (CoQ10) supplementation appears to be associated with a lower blood pressure. Nevertheless, it remains unclear whether food-sourced CoQ10 will affect new-onset hypertension in general adults. This study investigated the relationship between dietary CoQ10 intake and new-onset hypertension among the general population. Participants without hypertension at baseline from the China Health and Nutrition Survey (CHNS) prospective cohort study were included (n = 11,428). Dietary CoQ10 intake was collected by validated dietary recalls and the food weighing method. Linear and non-linear relationships between dietary CoQ10 intake and new-onset hypertension were analyzed using multivariable Cox proportional hazards models and restricted cubic splines. During follow-up (median: 6 years), 4006 new-onset hypertension cases were documented. Compared with non-consumers, the hazard ratio (HR) and 95% confidence interval (CI) from quintile 2 to 4 total dietary CoQ10 were 0.83 (0.76, 0.91), 0.86 (0.78, 0.94) and 1.01 (0.92, 1.11); total plant-derived CoQ10 were 0.80 (0.73, 0.88), 1.00 (0.91, 1.09) and 1.10 (1.00, 1.20); and animal-derived CoQ10 were 0.65 (0.59, 0.71), 0.58 (0.53, 0.64) and 0.68 (0.62, 0.75). The lowest risk was found at moderate intake, with a non-linear relationship (*P* nonlinearity < 0.05). Furthermore, the overall inverse association was stronger among individuals without alcohol consumption or eating a low-fat diet. Moderate long-term dietary CoQ10 intake might be protective against new-onset hypertension. However, it follows a non-linear relationship and excessive intake may increase the risk of new-onset hypertension in the Chinese population.

## 1. Introduction

Hypertension, the primary modifiable risk factor for cardiovascular disease (CVD) and its related morbidity and mortality (accounting for 10.8 million deaths annually), persists as a growing public health concern worldwide [1,2]. The prevalence of hypertension has increased at an alarming rate globally, doubling over the past 30 years, with an even more substantial increase in developing countries [2,3]. Proper hypertension management is crucial for reducing its related CVD risks and associated mortality. Given that dietary habits are key modifiable factors for hypertension, this is a promising prevention strategy to reverse the ever-rising prevalence of hypertension [1,4,5]. 

Coenzyme Q10 (CoQ10) is a lipid-soluble quinone with a central benzoquinone ring. It can be supplemented exogenously through CoQ10 supplements or a variety of foods [6]. Its principal role in the cell is to participate in the electron transport chain in the inner mitochondrial membrane, acting as a cofactor in the synthesis of ATP [6]. Additionally, as a crucial antioxidant, CoQ10 protects both mitochondrial and extra-mitochondrial cellular membranes against oxidative stress [7]. It also helps maintain lysosomal pH and participates in the metabolism of pyrimidines, sulfides, and amino acids [8]. 

Previous animal and human studies have demonstrated that CoQ10 deficiency is associated with many chronic diseases, including hypertension, diabetes and dyslipidemia [9]. Hitherto, prior human studies limited to small-scale short-term randomized controlled trials (RCTs) have evaluated various high doses of CoQ10 supplementation on surrogate markers of CVD risks, such as blood pressure [10]. Nevertheless, the widespread availability of CoQ10 supplements is hindered by their high cost, posing a challenge for general population access. Consequently, the efficacy of dietary sources of CoQ10 in the management of blood pressure carries substantial public health significance. However, there has been no prospective investigation of the relationship between long-term dietary CoQ10 intake and new-onset hypertension in the general population of adults. 

Therefore, using the nationwide China Health and Nutrition Survey (CHNS) covering 15 provinces, we prospectively investigated the potential association and dose–response relationships between long-term total, plant and animal source dietary CoQ10 intake and new-onset hypertension in the general adults. Additionally, we identified sub-populations that may derive the most benefit from dietary CoQ10 intake.

## 2. Materials and Methods

### 2.1. Study Design and Population

The CHNS study started in 1989 and has been followed up every 2–3 years since then. The study seeks to determine how social and economic change affects the health-related outcomes and nutritional status of Chinese people. Participants from nine provinces in 1997 and 3 large autonomous cities in China in 2011 were selected by a multistage, random cluster approach. A total of 11 waves of data collection have been conducted to date [11]. Surveys were conducted by well-trained interviewers among all members of the selected households. The comprehensive study design and primary findings have been delineated in another publication [12].

Seven waves of data from 1997 to 2015 were used in this study. Among the 27,887 eligible participants, those under 18 years old, pregnant or suffering from stroke, myocardial infarction, or any type of tumor at baseline were excluded. Among the remaining participants, those without BP data or those with hypertension (defined as having a systolic blood pressure [SBP] exceeding 140 mm Hg and/or a diastolic blood pressure [DBP] exceeding 90 mm Hg at baseline, prior physician diagnosis, or currently being treated with an antihypertensive medication) were excluded from the study. Further, we excluded participants with less than two survey rounds, missing dietary CoQ10 data, or extreme dietary energy intake (men: <800 or >8000 kcal/d; women: <600 or >6000 kcal/d). Finally, 11,428 participants made up the total sample (Additional file: Figure S1).

This study received approval from the institutional review committees at the University of North Carolina and the National Institute of Nutrition and Food Safety, Chinese Center for Disease Control and Prevention. Written informed consent was obtained from all of the participants.

### 2.2. Dietary Nutrient Intake

Data collection occurred at both the individual and household levels during each survey round. Specifically, three consecutive validated 24 h dietary recalls [13] were conducted by a trained investigator. Within each sampling unit, three consecutive days were randomly chosen from Monday to Sunday, ensuring nearly equal distribution across the week. Cooking oil and condiment consumption were determined by examining household inventory changes over the same three days and weighing them accordingly. Nutrient intake was calculated using the Chinese Food Composition Tables [14,15,16] by multiplying the consumed volume by the nutrient content in per standard portion size (100 g), and the total nutrient intake was aggregated across all food items. The CoQ10 intakes were calculated based on previous studies about coenzyme Q10 content in foods [6]. The detailed methodologies for dietary measurements in the CHNS have been previously documented [17]. 

To enhance the representation of long-term intake and reduce intra-individual variation, cumulative average intake values for each nutrient were computed using data up to the last visit before the occurrence of new-onset hypertension [18].

### 2.3. Assessment of Covariates

Blood pressure was measured by trained research staff using a mercury manometer after five minutes of rest, following the standard method at each follow-up. The mean of three measurements taken on the same arm was recorded in a well-lit, quiet room.

During each follow-up survey, questionnaires were used to collect information on age, sex, residence (urban or rural), education level, and smoking and drinking status. Smoking was defined as the use of cigarettes, including hand-rolled or device-rolled, and drinking was defined as drinking alcohol consumption. Height and weight were measured using calibrated equipment according to standard procedures. Body mass index (BMI) was calculated by dividing weight in kilograms by height in meters squared, in accordance with the World Health Organization (WHO) definition [19].

### 2.4. Study Outcome

New-onset hypertension was defined as the following three criteria: mean SBP ≥ 140 mmHg or mean diastolic BP of ≥90 mmHg (According to the Guidelines for Prevention and Treatment of Hypertension in China, Revised Edition in 2018) or diagnosed by a physician or under antihypertensive therapy during the follow-up.

Whenever a participant was first diagnosed with new-onset hypertension, the follow-up time was calculated as the mid-point between this diagnosis and the previous survey. For participants who did not develop hypertension in any subsequent surveys, the follow-up time was determined based on the date of the most recent survey.

### 2.5. Statistical Analysis

Baseline characteristics are presented as mean (standard deviation) or median (interquartile range) for continuous variables, and as percentages for categorical variables, based on quartiles of absolute dietary CoQ10. ANOVA tests or chi-square tests were employed to compare population characteristics across quartiles of dietary CoQ10 intake. Person-years of follow-up for each participant were calculated from the baseline questionnaire return date to the first hypertension diagnosis or the end of follow-up, whichever occurred first. 

For the primary analysis, we utilized restricted cubic splines with Cox proportional hazards models to explore potential nonlinear relationships between dietary CoQ10 consumption and the risk of new-onset hypertension. The Cox proportional hazards models estimated hazards ratio (HR) and 95% confidence interval (95% CI) for the association between dietary CoQ10 intake and risk of new-onset hypertension. The models were adjusted for baseline age (continuous), sex (male or female), BMI (continuous), education level (middle school or below, high school or college or above), smoking (yes or no), drinking status (yes or no), physical activity (continuous), residence (urban or rural), marital status (yes or no), and cumulative average intake of vegetables (continuous), fruits (continuous), and energy (continuous). 

Analyses were stratified by sex (male or female), age (<45 or ≥45 years), BMI (<22.1 or ≥22.1 [median] kg/m^2^), smoking status (no or yes), drinking status (no or yes), SBP (<120 or ≥120 mmHg), urban residence (no or yes), fat intake (<62.9 or ≥62.9 [median] g/day), carbohydrate intake (<312.8 or ≥312.8 [median] g/day) and protein intake (<65.2 or ≥65.2 [median] g/day). The multiplicative interaction effects were assessed using likelihood ratio tests of Cox proportional hazards models with and without the interaction terms.

The robustness of the primary findings was assessed through various sensitivity analyses. First, to reduce the potential impact of reverse causality, a latency analysis was performed by associating dietary CoQ10 intake with new-onset hypertension occurring two years after the reported intake. Second, follow-up person-time was calculated from baseline to the initial hypertension diagnosis. Third, to control for total energy intake as a confounder, analyses were repeated using the energy-adjusted residual method for dietary CoQ10 intakes [20]. Fourth, missing covariate values were addressed using multiple imputation with multivariate imputation by chained equation, followed by a sensitivity analysis of dietary CoQ10 on new-onset hypertension using complete data. The number of missing covariates is detailed in Additional file: Table S1. Fifth, to explore whether the results varied with different dietary CoQ10 intake categorizations, the analysis was conducted by dividing dietary CoQ10 into three groups (≤3.23, 3.23 to ≤5.52 mg/day, and >5.52 mg/day) and five groups (≤2.36, 2.36 to ≤3.64 mg/day, 3.64 to ≤4.96 mg/day, 4.96 to ≤7.04 mg/day, and >7.04 mg/day).

The statistical significance was set at 0.05 for 2-sided tests. All analyses were conducted utilizing R software version 4.1.3 (http://www.R-project.org/).

## 3. Results

### 3.1. Characteristics of the Study Participants

We included 11428 participants (45.7% male) with an average age of 41.7 years (SD, 13.9 years). The median total dietary CoQ10, plant- and animal-derived CoQ10 intake were 4.3 [2.7; 6.4], 2.1 [0.9; 3.9], and 1.7 [0.7; 2.8] mg/day, respectively.

Baseline characteristics by CoQ10 intake quartiles and sex are detailed in Table 1 and Additional file: Table S2, respectively. Participants with higher dietary CoQ10 intake were more likely to be male; had higher BMI, SBP, DBP, and education levels; had higher smoking and alcohol drinking rates; were married; resided in urban areas; and were less physically active. They also consumed more energy, fat, protein and vegetables, with lower carbohydrate consumption. During a median follow-up of 6 years (IQR: 4–13 years), 4006 participants (92,087 person-years) documented new-onset hypertension.

### 3.2. Association between Dietary CoQ10 Intake and New-Onset Hypertension

**Figure 1:**
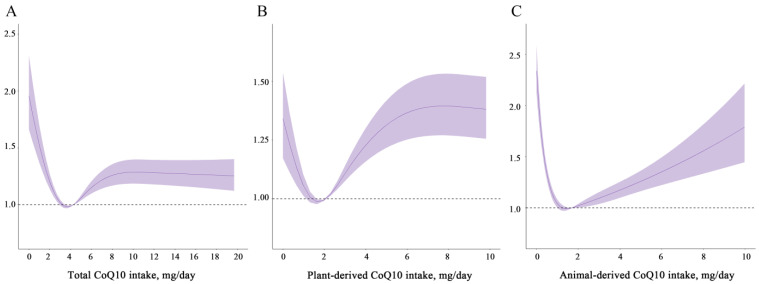
Restricted cubic spline models for the relationship between dietary CoQ10 intake and new-onset hypertension. (**A**) Total, (**B**) plant-derived, (**C**) animal-derived dietary CoQ10. Adjusted for baseline age (continuous), sex (male/female), BMI (continuous), education (middle school or below/high school/college or above), smoking status (yes/no), drinking status (yes/no), physical activity (continuous), residence (urban/rural), marital status (yes/no), and cumulative average intake of energy (continuous), vegetables (continuous), and fruits (continuous). Solid lines indicate point estimates, while ribbons denote 95% CIs. Abbreviations: BMI (body mass index), CI (confidence interval).

After adjusting for potential confounders, HR (95% CI) from low to high dietary total CoQ10 quintiles were: 1 (reference), 0.83 (0.76, 0.91), 0.86 (0.78, 0.94) and 1.01 (0.92, 1.11); plant-derived dietary CoQ10 were: 1 (reference), 0.80 (0.73, 0.88), 1.00 (0.91, 1.09) and 1.10 (1.00, 1.20); animal-derived dietary CoQ10 were: 1 (reference), 0.65 (0.59, 0.71), 0.58 (0.53, 0.64) and 0.68 (0.62, 0.75) (Table 2). In the cubic spline model adjusted for the same confounders, the relationship between total dietary CoQ10 and new-onset hypertension was non-linear (*p* < 0.05 for non-linearity). As CoQ10 consumption increased, the steepness of the slope decreased until a minimal risk was observed (Figure 1A). In addition, a similar trend was seen for plant-derived CoQ10, and there was a U-shaped association between animal-derived CoQ10 and new-onset hypertension (all *p* for non-linearity < 0.001, Figure 1B,C).

### 3.3. Subgroup Analyses by Potential Effect Modifiers

**Figure 2:**
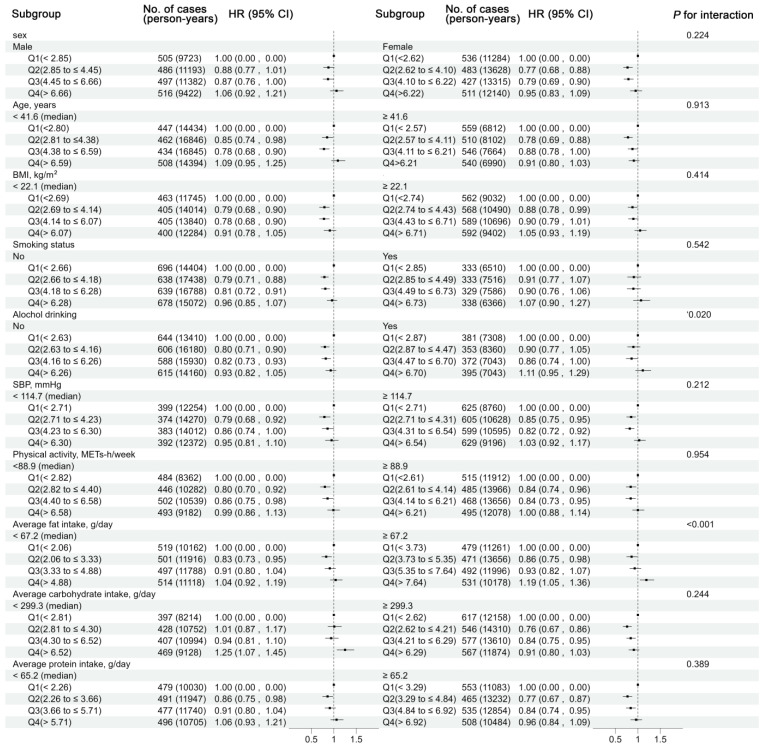
Stratified analyses by potential effect modifiers for the association between dietary CoQ10 intake and new-onset hypertension in various subgroups. Adjusted, but not stratified, for baseline age (continuous), sex (male/female), BMI (continuous), education (middle school or below, high school or college or above), smoking status (yes/no), drinking status (yes/no), physical activity (continuous), residence (urban/rural), marital status (yes/no), and cumulative average intake of energy (continuous), vegetables (continuous), and fruits (continuous). The likelihood ratio test was used to calculate the *P* interaction. Abbreviations: BMI (body mass index), CI (confidence interval), HR (hazard ratio), SBP (systolic blood pressure).

**Figure 3:**
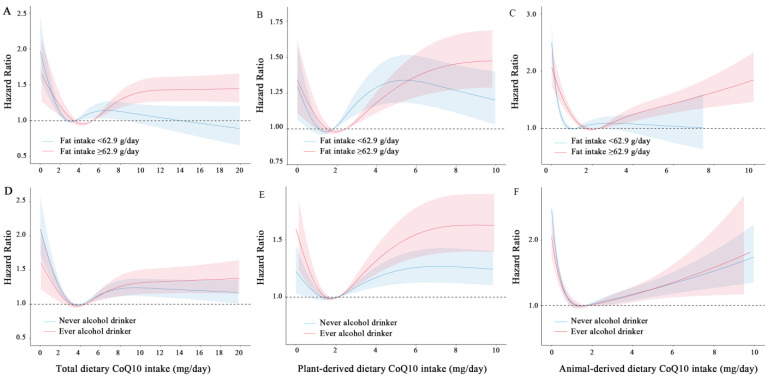
Restricted cubic spline models for the relationship between dietary CoQ10 intake and new-onset hypertension stratified by average dietary fat intake and baseline drinking status. (**A**) Total, (**B**) plant-derived, (**C**) animal-derived dietary CoQ10 stratified by average dietary fat intake. (**D**) Total, (**E**) plant-derived, (**F**) animal-derived dietary CoQ10 stratified by baseline drinking status. Adjusted, if not stratified, for at baseline age (continuous), sex (male/female), BMI (continuous), education (middle school or below/high school/college or above), smoking status (yes/no), drinking status (yes/no), physical activity (continuous), residence (urban/rural), marital status (yes/no), and cumulative average intake of energy (continuous), vegetables (continuous), and fruits (continuous). The likelihood ratio test was used to calculate the *P* interaction.

The relationship between dietary CoQ10 intake and the risk of new-onset hypertension was analyzed across different subgroups. Baseline alcohol consumption and a high-fat diet significantly modulated this association (*P* _interaction_ < 0.05; Figure 2). Specifically, an inverse association between moderate total dietary CoQ10 intake and incident hypertension was present in participants without alcohol consumption at baseline, while this was not present among participants with alcohol consumption (*P*_interaction_ = 0.020, Figure 2 and Figure 3). Alcohol-free participants with moderate total dietary CoQ10 intake had a 18–20% lower risk of new-onset hypertension compared with those with the lowest dietary CoQ10 intake [HR (95% CI) for Q2 vs. Q1: 0.80 (0.71, 0.90); Q3 vs. Q1: 0.82 (0.73, 0.93)], whereas in participants with alcohol consumption, moderate total dietary CoQ10 intake did not show any protective effects [Q2 vs. Q1: 0.90 (0.77, 1.05); Q3 vs. Q1: 0.86 (0.74, 1.00)]. 

Participants in both low- and high-fat intake groups were observed to be at lower risk in the second quintile after multivariable adjustments [1.04 (0.92, 1.19) and 1.19 (1.05, 1.36)] (Figure 2 and Figure 3). Furthermore, higher dietary CoQ10 intake was associated with a lower risk of new-onset hypertension in participants with low-fat diets (<62.9 g/day) than those with high-fat diets (≥62.9 g/day) (Figure 3). A total CoQ10 intake of more than 7.64 mg per day was associated with a 19% higher risk of new-onset hypertension in participants with a high-fat diet [1.19 (1.05–1.36)], while there was no higher risk in participants with a low-fat diet [1.04 (0.92–1.19); Figure 2]. The results did not vary across the other subgroups.

### 3.4. Sensitivity Analyses

The results of RCS remained generally consistent with the overall findings when a 2-year lag was included. Follow-up person-time was calculated from baseline until the first hypertension diagnosis using a multiple imputation procedure with energy-adjusted residues of dietary CoQ10 intake (Additional file: Figure S2).

Sensitivity analyses showed no substantial influence on the original results from the multivariable Cox regression model when we excluded hypertension cases occurring within the first 2 years of follow-up. Calculating follow-up person-time for new-onset hypertension from baseline until the first diagnosis and the nearest survey before did not significantly alter the findings, when using energy-adjusted CoQ10 intake and the risk of new-onset hypertension, imputing missing values using multiple imputation with chained equations, and dividing dietary CoQ10 intake into three or five (Additional file: Tables S3–S8). 

## 4. Discussion

In this large, national, longitudinal cohort study, we initially investigated the association between dietary CoQ10 and new-onset hypertension among general Chinese adults. The intake of dietary CoQ10 (total, plant- and animal-derived CoQ10) demonstrated a non-linear relationship with the risk of new-onset hypertension. An appropriate intake of dietary CoQ10 was associated with a decreased risk of new-onset hypertension. Furthermore, we found that the inverse association was more pronounced in individuals who abstained from alcohol at baseline and consumed low-fat diets. 

Existing small-scale clinical trials have investigated the effect of CoQ10 supplementation on intermediate indicators of blood pressure. Although the findings are not yet consistent, they suggest a potential link between them. Two intervention studies conducted in healthy population indicated that high doses of CoQ10 (900 mg/day) over a duration of 4 weeks did not have a significant impact on BP [21,22]. While focusing on the cardiometabolic diseases population, our recent dose–response meta-analysis of twenty-six RCTs with durations ranging from 1 to 24 months showed that CoQ10 supplementation significantly decreased SBP (WMD: −4.77 mmHg; 95% CI: −6.57 to −2.97) with a clinically optimal dose of 100–200 mg/day [10]. Nonetheless, the doses of CoQ10 used in these trials were significantly higher than those typically consumed from dietary food by the general population. Dietary CoQ10, readily accessible through daily nutrition, carries greater public health significance. However, its long-term effect on new-onset hypertension requires investigation through prospective cohort studies. Furthermore, its high cost as a supplement limits its widespread use for preventing hypertension in the general population. 

In our current study, the daily median dietary CoQ10 intake typically aligns with the population in Denmark, with values ranging from 3 to 5 mg/day [23], 5.4 mg/day for men and 3.8 mg/day for women in Finland [24], and approximately 4.48 mg/day in Japan (4.48 mg/day for total CoQ10) [25], primarily derived from animal foods (64% of the daily intake), such as meat, poultry or eggs, while plant foods, such as cereals, fruit, edible fats, and vegetables only make minor contributions [26]. 

Our study demonstrated that moderate CoQ10 intake (2.71–6.39 mg/day in Q2–3 consumption groups) was associated with a lower risk of new-onset hypertension compared with the lowest quartiles. CoQ10 can be obtained through dietary intake and synthesized endogenously in the human body. Blood levels of CoQ10 exhibit considerable variability in the general population, influenced by factors such as age, health status, and medication use. However, due to methodological limitations of the CHNS program, we were unable to measure blood CoQ10 levels directly. Nevertheless, we controlled for numerous confounding factors to minimize their potential impact. These findings are consistent with our recent cohort study, which demonstrated a negative association between moderate CoQ10 intake from diverse dietary sources and the incidence of new-onset hypertension [27].

It is noteworthy that there are non-linear associations between total, plant- and animal-derived dietary CoQ10 intake and the incidence of new-onset hypertension in general adults. As the intake of dietary CoQ10 increased, the risk of new-onset hypertension gradually decreased. When the intake exceeded approximately 4 mg per day, the risk of new-onset hypertension showed a slight upward trend before reaching a plateau. Long-term appropriate intake of dietary CoQ10 may help maintain the stability and persistence of serum CoQ10, potentially lowering the risk of new-onset hypertension. Similar to total dietary CoQ10, plant-derived CoQ10 also demonstrated a nonlinear relationship, with moderate intake being inversely associated with the risk of new-onset hypertension. Previous studies suggested plant-based foods can reduce the risk of hypertension, likely due to their beneficial components such as vitamins, antioxidants and fiber [28,29]. Animal foods, which are the primary dietary CoQ10 contributors (e.g., beef heart, animal liver, chicken), exhibit a U-shaped association between CoQ10 content and the risk of new-onset hypertension. When the intake of animal-derived CoQ10 exceeded approximately 1.5 mg per day, higher levels significantly increased the risk of new-onset hypertension. Practically, consuming more than 15 g of red meat daily (which contains 16.1–36.5 mg/kg of CoQ10) reaches this threshold, while the average daily red meat intake for Chinese adults is 55.1 g [30]. It should be kept in mind that several animal food ingredients (such as advanced glycation end products, branched amino acids, heterocyclic aromatic amines, nitrate, and bioactive compounds) have been linked to negative health consequences [31,32]. There is also evidence that red meat contains L-carnitine, a trimethylamine, which can be metabolized by the gut microbiota to produce trimethylamine-N-oxide, increasing the risk of atherosclerosis in rodents [33]. Similar to this study, a meta-analysis that included seven studies showed a positive association between the risk of hypertension and red meat intake compared to the lowest intake group (risk ratios (RR): 1.08, 95% CI: 1.06, 1.11) [34]. Additionally, our recent cohort study indicated that the intake of CoQ10 from red meat also exhibited a U-shaped nonlinear dose-response relationship with new-onset hypertension. Compared to the lowest quantile, the highest quantile intake has an HR of 1.25 (1.11, 1.41) [27]. Therefore, it would be advisable to contemplate the inclusion of supplemental CoQ10 to attain the intended preventive or therapeutic effects.

Hitherto, a number of dietary approaches extensively reported, such as Dietary Approaches to Stop Hypertension (DASH) and Mediterranean diets, etc., have been extensively studied for their beneficial effects on blood pressure regulation. Specifically, an intervention study on Mediterranean diets demonstrated a significant reduction in systolic blood pressure (−5.5 mm Hg; 95% CI, −10.7 to −0.4), which may be attributed to its antioxidant-rich components from fruits, vegetables, and olive oil [35,36]. Furthermore, it was reported that dietary diversity has been associated with improved blood pressure, especially with some healthier rich-CoQ10 choices [27]. Our study indicated that increased CoQ10 intake from dietary sources also means a higher intake of other nutrients (such as fats, potentially posing health risks) [37]. Chinese heart-healthy diets (CHH), which integrate diverse elements of Chinese cuisine, contribute to increased food variety and significant reductions in SBP (−5.0 mm Hg, 95% CI, −6.5 to −3.5) and DBP (−2.8 mm Hg; 95% CI, −3.7 to −1.9) [38]. Therefore, this current study suggests consuming healthier CoQ10-rich foods such as olive oil, seafood, broccoli, and soybeans, and limiting the intake of red meat or organ meats. Our study supports broader dietary guidelines aimed at reducing new-onset hypertension through healthier food choices.

A multi-factorial mechanism may be involved in CoQ10 intake preventing hypertension. It is well known that the hypotensive properties of CoQ10 may be largely attributed to its antioxidant properties [39], which enhance NO bioavailability, exerting a direct beneficial effect on the endothelium [40]. Besides, it exerts angiotensin-like effects in sodium retention and anti-inflammatory effects [41,42]. Various studies indicate that CoQ10 mediates its beneficial effects through both direct and indirect anti-inflammatory mechanisms. Although several previous animal studies and RCTs have suggested that CoQ10 supplements may reduce inflammation [43,44], our recently published study demonstrated a negative association between dietary CoQ10 and hsCRP levels, with an L-shaped dose–response relationship [45]. The precise mechanism is yet to be elucidated through further investigation.

It is interesting to note that moderate dietary CoQ10 intake exerts greater benefits for those who do not consume alcohol than for those who consume alcohol at baseline. The beneficial effects of CoQ10 may be partially offset by the detrimental effect of alcohol consumption on blood pressure, nitric oxide bioavailability, endothelial function, platelet function, thrombosis, blood lipids and inflammation [46]. Mechanistic studies have demonstrated that ethanol-induced CoQ10 depletion may mediate cytotoxicity in HepG2 cells [9,47]. These findings provide evidence that moderate CoQ10-rich foods might mitigate the risk of new-onset hypertension in non-alcohol consumers. Moreover, individuals with high-fat diets tend to have higher CoQ10 intake compared to those with low-fat diets. On the one hand, a high-fat diet contributes to the elevation of blood pressure and increases the risk profile as previously noted [48,49,50]. On the other hand, high fat consumption appeared to be associated with unfavorable changes in gut microbiota and fecal metabolomic profiles [51], which might need more CoQ10 to attenuate its detrimental effects. These findings emphasized the necessity of maintaining moderate intakes of dietary CoQ10 and provided confirmatory evidence that nutritional guidelines should advise against increasing intake of dietary fat. Taken together, these findings highlight the potential of achieving primary prevention of hypertension by ensuring appropriate consumption of CoQ10-rich foods, especially among high-risk individuals.

The primary strength of this study is that it addresses a critical knowledge gap regarding what intake of food source CoQ10 is required to achieve maximum benefit in the prevention of new-onset hypertension through extensive adjustments of covariables based on a prospective cohort design. In addition, repeated and validated measurements of three-day dietary records enabled us to calculate the cumulative average of exposure to dietary factors. Lastly, detailed subgroup analyses were conducted to evaluate the consistency of the results, along with sensitivity analyses to assess the robustness of the findings.

Limitations also need to be considered. First, as dietary CoQ10 intake cannot be accurately estimated, a more credible approach to identifying dietary CoQ10 exposure is needed to confirm these associations. Future research should investigate more reliable methods to accurately measure CoQ10 contents in food, thereby enhancing the validity of the associations identified in our study. Second, no data were available on the levels of circulating CoQ10. As a result, the relationship between circulating CoQ10 and new-onset hypertension could not be examined. It is essential for future studies to include measurements of circulating CoQ10 levels to better understand its physiological impact and validate dietary intake assessments. Third, the CHNS cohort was racially homogeneous, limiting its generalizability. Future studies including a broader range of ethnic and demographic groups are warranted to verify our findings. Finally, considering that observational study designs naturally carry the risk of potential residual confounding and the potential for reverse causation, it is advisable to approach the interpretation of the findings with caution.

## 5. Conclusions

Our study suggests a non-linear relationship between dietary sources of CoQ10 and the risk of new-onset hypertension in general Chinese adults. Appropriate dietary CoQ10 intake seems to provide protection against new-onset hypertension, whereas excessive intake increases the risk.

## Figures and Tables

**Table 1:** Baseline population characteristics by quintiles of dietary CoQ10 intake ^1^.

Characteristics	Dietary CoQ10 Intake (mg/day)					*p*
	Total	Q1 (≤2.71)	Q2 (2.71 to ≤4.26)	Q3 (4.26 to ≤6.39)	Q4 (>6.39)	
Participants, *n*	11428	2857	2857	2857	2857	-
Age, years	41.7 (13.9)	42.8 (14.9)	41.9 (13.7)	41.2 (13.5)	40.9 (13.4)	<0.001
Man, (*n* %)	5220 (45.7%)	1204 (42.1%)	1243 (43.5%)	1371 (48.0%)	1402 (49.1%)	<0.001
BMI, kg/m^2^	22.5 (3.2)	22.4 (3.3)	22.2 (3.1)	22.4 (3.1)	22.8 (3.2)	<0.001
SBP, mmHg	114.1 (11.5)	113.9 (11.6)	113.7 (12.0)	114.0 (11.4)	114.8 (10.8)	0.002
DBP, mmHg	74.2 (7.8)	73.8 (8.0)	73.7 (8.1)	74.2 (7.6)	75.2 (7.5)	<0.001
Education level, (*n* %)						<0.001
Middle school or below	7912 (70.6%)	2081 (74.7%)	2008 (71.6%)	1920 (68.3%)	1903 (67.9%)	
High school	2452 (21.9%)	507 (18.2%)	612 (21.8%)	662 (23.6%)	671 (23.9%)	
College or above	838 (7.5%)	197 (7.1%)	183 (6.5%)	228 (8.1%)	230 (8.2%)	
Former or current smoker, (*n* %)	3363 (29.6%)	773 (27.2%)	790 (27.7%)	875 (30.8%)	925 (32.5%)	<0.001
Alcohol consumer, (*n* %)	3918 (34.7%)	876 (30.9%)	951 (33.7%)	1012 (36.0%)	1079 (38.1%)	<0.001
Married, (*n* %)	9544 (84.2%)	2327 (82.3%)	2414 (85.1%)	2372 (83.8%)	2431 (85.8%)	<0.001
Urban residence, (*n* %)	4396 (38.5%)	967 (33.8%)	1071 (37.5%)	1166 (40.8%)	1192 (41.7%)	<0.001
Physical activity, METs-h/week, median [IQR]	88.9 [18.0; 206.4]	99.6 [18.6; 227.5]	92.8 [18.1; 217.4]	86.0 [17.7; 193.2]	82.2 [17.1; 192.0]	<0.001
Energy, kcal/d	2144.3 (564.0)	1988.6 (568.8)	2084.9 (518.0)	2187.3 (528.8)	2316.4 (584.9)	<0.001
Total fat, % of energy	29.8 (10.5)	24.7 (11.0)	28.6 (9.3)	31.2 (9.1)	34.5 (9.8)	<0.001
Total carbohydrate, % of energy	56.8 (11.3)	62.3 (12.0)	57.9 (10.2)	55.1 (9.8)	51.9 (10.3)	<0.001
Total protein, % of energy	12.7 (2.7)	12.4 (2.8)	12.8 (2.6)	13.0 (2.6)	12.8 (2.9)	<0.001
Vegetable intake, g/day	301.1 (136.3)	279.9 (141.5)	303.0 (127.3)	313.6 (133.4)	307.9 (140.1)	<0.001
Fruit intake, g/day	0.0 [0.0; 50.0]	0.0 [0.0; 25.0]	0.0 [0.0; 41.7]	0.0 [0.0; 57.5]	0.0 [0.0; 83.3]	<0.001

^1^ Continuous data are reported as mean (SD), median (IQR), while categorical data are shown as n (%). Baseline data on nondietary factors were collected, and dietary data were calculated as cumulative average from baseline and follow-up periods. Abbreviations: BMI (body mass index), DBP (diastolic blood pressure), IQR (indicates interquartile range), SBP (systolic blood pressure).

**Table 2:** The relationship of dietary CoQ10 intake with risk of new-onset hypertension.

CoQ10 Intake, mg/Day	No. of Cases (Person-Years)	Crude Model		Adjusted Model ^1^	
		HR (95% CI)	*p*	HR (95% CI)	*p*
Total					
Q1 (≤2.71)	1023 (21,004)	Ref	-	Ref	-
Q2 (2.71 to ≤4.26)	969 (25,012)	0.79 (0.72, 0.86)	<0.001	0.83 (0.76, 0.91)	<0.001
Q3 (4.26 to ≤6.39)	982 (24,568)	0.82 (0.75, 0.89)	<0.001	0.86 (0.78, 0.94)	0.001
Q4 (>6.39)	1032 (21,504)	0.99 (0.91, 1.08)	0.800	1.01 (0.92, 1.11)	0.800
Plant-derived					
Q1 (≤0.67)	915 (19,698)	Ref	-	Ref	
Q2 (0.67 to ≤1.69)	949 (26,948)	0.75 (0.68, 0.82)	<0.001	0.80 (0.73, 0.88)	<0.001
Q3 (1.69 to ≤2.81)	1046 (23,334)	0.97 (0.88, 1.06)	0.500	1.00 (0.91, 1.09)	>0.900
Q4 (>2.81)	1096 (22,107)	1.07 (0.98, 1.17)	0.130	1.10 (1.00, 1.20)	0.057
Animal-derived					
Q1 (≤0.94)	1229 (20,904)	Ref	-	Ref	
Q2 (0.94 to ≤2.07)	961 (24,248)	0.67 (0.61, 0.73)	<0.001	0.65 (0.59, 0.71)	<0.001
Q3 (2.07 to ≤3.93)	880 (24,675)	0.60 (0.55, 0.65)	<0.001	0.58 (0.53, 0.64)	<0.001
Q4 (>3.93)	936 (22,259)	0.71 (0.65, 0.77)	<0.001	0.68 (0.62, 0.75)	<0.001

^1^ Adjusted for Adjusted for baseline age (continuous), sex (male/female), BMI (continuous), education (middle school or below/high school/college or above), smoking status (yes/no), drinking status (yes/no), physical activity (continuous), residence (urban/rural), marital status (yes/no), and cumulative average intake of energy (continuous), vegetables (continuous), and fruits (continuous). Abbreviations: BMI, body mass index; HR, Hazard Ratio; CI, Confidence Interval.

## Data Availability

The original datasets are accessible on the CHNS official website (https://www.cpc.unc.edu/projects/china) (accessed on 10 April 2023).

## Supplementary Materials

The following supporting information can be downloaded at: https://www.mdpi.com/article/10.3390/nu16152478/s1, Figure S1: Flow chart; Figure S2: Dose-response relationship between dietary CoQ10 intake and new-onset hypertension across sensitivity analyses; Table S1: Missing covariates; Table S2: Participants’ characteristics by sex; Table S3: Association between dietary CoQ10 intake and new-onset hypertension, excluding cases diagnosed within the first two years of follow-up; Table S4: Association between dietary CoQ10 intake and new-onset hypertension, with follow-up person-time calculated from baseline to initial diagnosis of hypertension; Table S5: Association between energy-adjusted residues of dietary CoQ10 intake and new-onset hypertension; Table S6: Association between dietary CoQ10 intake and new-onset hypertension using a multiple imputation procedure with five rounds of imputation; Table S7: Relationship between dietary CoQ10 intake in three equal groups and risk of new-onset hypertension; Table S8: Relationship between dietary CoQ10 intake in five equal groups and risk of new-onset hypertension.

## References

[1] Schutte A.E., Srinivasapura Venkateshmurthy N., Mohan S., Prabhakaran D. (2021). Hypertension in Low- and Middle-Income Countries. Circ. Res..

[2] (2020). Global burden of 87 risk factors in 204 countries and territories, 1990–2019: A systematic analysis for the Global Burden of Disease Study 2019. Lancet.

[3] Mills K.T., Stefanescu A., He J. (2020). The global epidemiology of hypertension. Nat. Rev. Nephrol..

[4] Kotchen T.A., Kotchen J.M., Boegehold M.A. (1991). Nutrition and hypertension prevention. Hypertension.

[5] Srinath Reddy K., Katan M.B. (2004). Diet, nutrition and the prevention of hypertension and cardiovascular diseases. Public Health Nutr..

[6] Pravst I., Zmitek K., Zmitek J. (2010). Coenzyme Q10 contents in foods and fortification strategies. Crit. Rev. Food Sci. Nutr..

[7] Pallotti F., Bergamini C., Lamperti C., Fato R. (2021). The Roles of Coenzyme Q in Disease: Direct and Indirect Involvement in Cellular Functions. Int. J. Mol. Sci..

[8] Mantle D., Lopez-Lluch G., Hargreaves I.P. (2023). Coenzyme Q10 Metabolism: A Review of Unresolved Issues. Int. J. Mol. Sci..

[9] Vidyashankar S., Nandakumar K.S., Patki P.S. (2012). Alcohol depletes coenzyme-Q(10) associated with increased TNF-alpha secretion to induce cytotoxicity in HepG2 cells. Toxicology.

[10] Zhao D., Liang Y., Dai S., Hou S., Liu Z., Liu M., Dong X., Zhan Y., Tian Z., Yang Y. (2022). Dose-response Effect of Coenzyme Q10 Supplementation On Blood Pressure Among Patients with Cardiometabolic Disorders: A GRADE-assessed Systematic Review and Meta-analysis of Randomized Controlled Trials. Adv. Nutr..

[11] Zhang B., Zhai F.Y., Du S.F., Popkin B.M. (2014). The China Health and Nutrition Survey, 1989–2011. Obes. Rev..

[12] Popkin B.M., Du S., Zhai F., Zhang B. (2010). Cohort Profile: The China Health and Nutrition Survey--monitoring and understanding socio-economic and health change in China, 1989–2011. Int. J. Epidemiol..

[13] Zhai F., Guo X., Popkin B.M., Ma L., Wang Q., Shuigao W.Y., Jin, Ge K. (1996). Evaluation of the 24-Hour Individual Recall Method in China. Food Nutr. Bull..

[14] Institute for Nutrition and Food Hygiene of the Chinese Academy of Preventive Medicine (1991). Food Composition Table.

[15] Institute for Nutrition and Food Hygiene of the Chinese Academy of Preventive Medicine (2002). Food Composition Table.

[16] Institute for Nutrition and Food Hygiene of the Chinese Academy of Preventive Medicine (2005). Food Composition Table.

[17] Zhao D., Tian Z., Liang Y., Chen H., Fan Z., Liu Z., Dai S., Liu M., Kuang H., Yang Y. (2022). J-Shaped Association of Tomato Intake with New-Onset Hypertension in General Adults: A Nationwide Prospective Cohort Study. Nutrients.

[18] Hu F.B., Stampfer M.J., Rimm E., Ascherio A., Rosner B.A., Spiegelman D., Willett W.C. (1999). Dietary fat and coronary heart disease: A comparison of approaches for adjusting for total energy intake and modeling repeated dietary measurements. Am. J. Epidemiol..

[19] https://www.who.int/news-room/fact-sheets/detail/obesity-and-overweight.

[20] Willett W., Stampfer M.J. (1986). Total energy intake: Implications for epidemiologic analyses. Am. J. Epidemiol..

[21] Ikematsu H., Nakamura K., Harashima S.-I., Fujii K., Fukutomi N. (2006). Safety assessment of coenzyme Q10 (Kaneka Q10) in healthy subjects: A double-blind, randomized, placebo-controlled trial. Regul. Toxicol. Pharmacol..

[22] Nukui K., Matsuoka Y., Yamagishi T., Miyawaki H., Sato K. (2007). Safety assessment of PureSorb-Q40 in healthy subjects and serum coenzyme Q10 level in excessive dosing. J. Nutr. Sci. Vitaminol..

[23] Weber C., Bysted A., Hølmer G. (1997). Coenzyme Q10 in the diet--daily intake and relative bioavailability. Mol. Aspects Med..

[24] Mattila P., Kumpulainen J. (2001). Coenzymes Q9 and Q10: Contents in foods and dietary intake. J. Food Compos. Anal..

[25] Kubo H., Fujii K., Kawabe T., Matsumoto S., Kishida H., Hosoe K. (2008). Food content of ubiquinol-10 and ubiquinone-10 in the Japanese diet. J. Food Compos. Anal..

[26] Weber C., Bysted A., Hłlmer G. (1997). The coenzyme Q10 content of the average Danish diet. Int. J. Vitam. Nutr. Res..

[27] Dai S., Tian Z., Zhao D., Liang Y., Zhong Z., Xu Y., Hou S., Yang Y. (2024). The Association between the Diversity of Coenzyme Q10 Intake from Dietary Sources and the Risk of New-Onset Hypertension: A Nationwide Cohort Study. Nutrients.

[28] Yannakoulia M., Scarmeas N. (2024). Diets. N. Engl. J. Med..

[29] Tomé-Carneiro J., Visioli F. (2023). Plant-Based Diets Reduce Blood Pressure: A Systematic Review of Recent Evidence. Curr. Hypertens. Rep..

[30] Du H., Guo Y., Bennett D.A., Bragg F., Bian Z., Chadni M., Yu C., Chen Y., Tan Y., Millwood I.Y. (2020). Red meat, poultry and fish consumption and risk of diabetes: A 9 year prospective cohort study of the China Kadoorie Biobank. Diabetologia.

[31] Lescinsky H., Afshin A., Ashbaugh C., Bisignano C., Brauer M., Ferrara G. (2022). Health effects associated with consumption of unprocessed red meat: A Burden of Proof study. Nat. Med..

[32] Wolk A. (2017). Potential health hazards of eating red meat. J. Intern. Med..

[33] Koeth R.A., Wang Z., Levison B.S., Buffa J.A., Org E., Sheehy B.T., Britt E.B., Fu X., Wu Y., Li L. (2013). Intestinal microbiota metabolism of l-carnitine, a nutrient in red meat, promotes atherosclerosis. Nat. Med..

[34] Schwingshackl L., Schwedhelm C., Hoffmann G., Knüppel S., Iqbal K., Andriolo V., Bechthold A., Schlesinger S., Boeing H. (2018). Food groups and risk of hypertension: A systematic review and dose-response meta-analysis of prospective studies. Adv. Nutr..

[35] Jennings A., Berendsen A.M., de Groot L.C.P.G.M., Feskens E.J.M., Brzozowska A., Sicinska E., Pietruszka B., Meunier N., Caumon E., Malpuech-Brugère C. (2019). Mediterranean-Style Diet Improves Systolic Blood Pressure and Arterial Stiffness in Older Adults. Hypertension.

[36] Filippou C., Thomopoulos C., Konstantinidis D., Siafi E., Tatakis F., Manta E., Drogkaris S., Polyzos D., Kyriazopoulos K., Grigoriou K. (2023). DASH vs. Mediterranean diet on a salt restriction background in adults with high normal blood pressure or grade 1 hypertension: A randomized controlled trial. Clin. Nutr..

[37] Oliveira M.C., Menezes-Garcia Z., Henriques M.C., Soriani F.M., Pinho V., Faria A.M., Santiago A.F., Cara D.C., Souza D.G., Teixeira M.M. (2013). Acute and sustained inflammation and metabolic dysfunction induced by high refined carbohydrate-containing diet in mice. Obesity (Silver Spring).

[38] Wang Y., Wang Y., Feng L., Zeng G., Zhu H., Sun J., Gao P., Yuan J., Lan X., Li S. (2022). Effects of Cuisine-Based Chinese Heart-Healthy Diet in Lowering Blood Pressure Among Adults in China: Multicenter, Single-Blind, Randomized, Parallel Controlled Feeding Trial. Circulation.

[39] Dai S., Tian Z., Zhao D., Liang Y., Liu M., Liu Z., Hou S., Yang Y. (2022). Effects of Coenzyme Q10 Supplementation on Biomarkers of Oxidative Stress in Adults: A GRADE-Assessed Systematic Review and Updated Meta-Analysis of Randomized Controlled Trials. Antioxidants.

[40] Flowers N., Hartley L., Todkill D., Stranges S., Rees K. (2014). Co-enzyme Q10 supplementation for the primary prevention of cardiovascular disease. Cochrane Database Syst. Rev..

[41] Langsjoen P., Willis R., Folkers K. (1994). Treatment of essential hypertension with coenzyme Q10. Mol. Aspects Med..

[42] Gasmi A., Bjørklund G., Mujawdiya P.K., Semenova Y., Piscopo S., Peana M. (2022). Coenzyme Q(10) in aging and disease. Crit. Rev. Food. Sci. Nutr..

[43] Fan L., Feng Y., Chen G.-C., Qin L.-Q., Fu C.-L., Chen L.-H. (2017). Effects of coenzyme Q10 supplementation on inflammatory markers: A systematic review and meta-analysis of randomized controlled trials. Pharmacol. Res..

[44] Sohet F.M., Neyrinck A.M., Pachikian B.D., de Backer F.C., Bindels L.B., Niklowitz P., Menke T., Cani P.D., Delzenne N.M. (2009). Coenzyme Q10 supplementation lowers hepatic oxidative stress and inflammation associated with diet-induced obesity in mice. Biochem. Pharmacol..

[45] Zhao M., Tian Z., Zhao D., Liang Y., Dai S., Xu Y., Hou S., Yang Y. (2023). L-shaped association between dietary coenzyme Q10 intake and high-sensitivity C-reactive protein in Chinese adults: A national cross-sectional study. Food Funct..

[46] Roerecke M., Kaczorowski J., Tobe S.W., Gmel G., Hasan O.S.M., Rehm J. (2017). The effect of a reduction in alcohol consumption on blood pressure: A systematic review and meta-analysis. Lancet Public Health.

[47] Loop R., Anthony M., Willis R., Folkers K. (1994). Effects of ethanol, lovastatin and coenzyme Q10 treatment on antioxidants and TBA reactive material in liver of rats. Mol. Aspects Med..

[48] Suffee N., Baptista E., Piquereau J., Ponnaiah M., Doisne N., Ichou F., Lhomme M., Pichard C., Galand V., Mougenot N. (2022). Impacts of a high-fat diet on the metabolic profile and the phenotype of atrial myocardium in mice. Cardiovasc. Res..

[49] Liu T., Wang B., Cao H. (2020). Effects of high-fat diet-induced gut microbiota dysbiosis: Far beyond the gut. Gut.

[50] Wilde D.W., Massey K.D., Walker G.K., Vollmer A., Grekin R.J. (2000). High-fat diet elevates blood pressure and cerebrovascular muscle Ca(2+) current. Hypertension.

[51] Wan Y., Wang F., Yuan J., Li J., Jiang D., Zhang J., Li H., Wang R., Tang J., Huang T. (2019). Effects of dietary fat on gut microbiota and faecal metabolites, and their relationship with cardiometabolic risk factors: A 6-month randomised controlled-feeding trial. Gut.

