# Supplementary material for: Associations between Long-Term Dietary Coenzyme Q10 Intake and New-Onset Hypertension in Adults: Insights from a Nationwide Prospective Cohort Study
Source: Nutrients. 2024 Jul 31;16(15):2478. doi: 10.3390/nu16152478 (PMC11313835; doi:10.3390/nu16152478)
Supplement: Supplementary file 1 [file nutrients-16-02478-s001.zip › nutrients-3104461-supplementary.pdf]

Supplemental Online Content

Supplemental Online Content 1
- Figure S1. Flow chart 2
- Figure S2. Dose-response relationship between dietary CoQ10 intake and new-onset hypertension across sensitivity analyses 3
- Table S1. Missing covariates 4
- Table S2. Participants characteristics by sex * 5
- Table S3. Association between dietary CoQ10 intake and new-onset hypertension, excluding cases diagnosed within the first two years of follow-up 6
- Table S4. Association between dietary CoQ10 intake and new-onset hypertension, with follow-up person-time calculated from baseline to initial diagnosis of hypertension 7
- Table S5. Association between energy-adjusted residues of dietary CoQ10 intake and new-onset hypertension 8
- Table S6. Association between dietary CoQ10 intake and new-onset hypertension using a multiple imputation procedure with five rounds of imputation 9
- Table S7. Relationship between dietary CoQ10 intake in three equal groups and risk of new-onset hypertension 10
- Table S8. Relationship between dietary CoQ10 intake in five equal groups and risk of new-onset hypertension 11

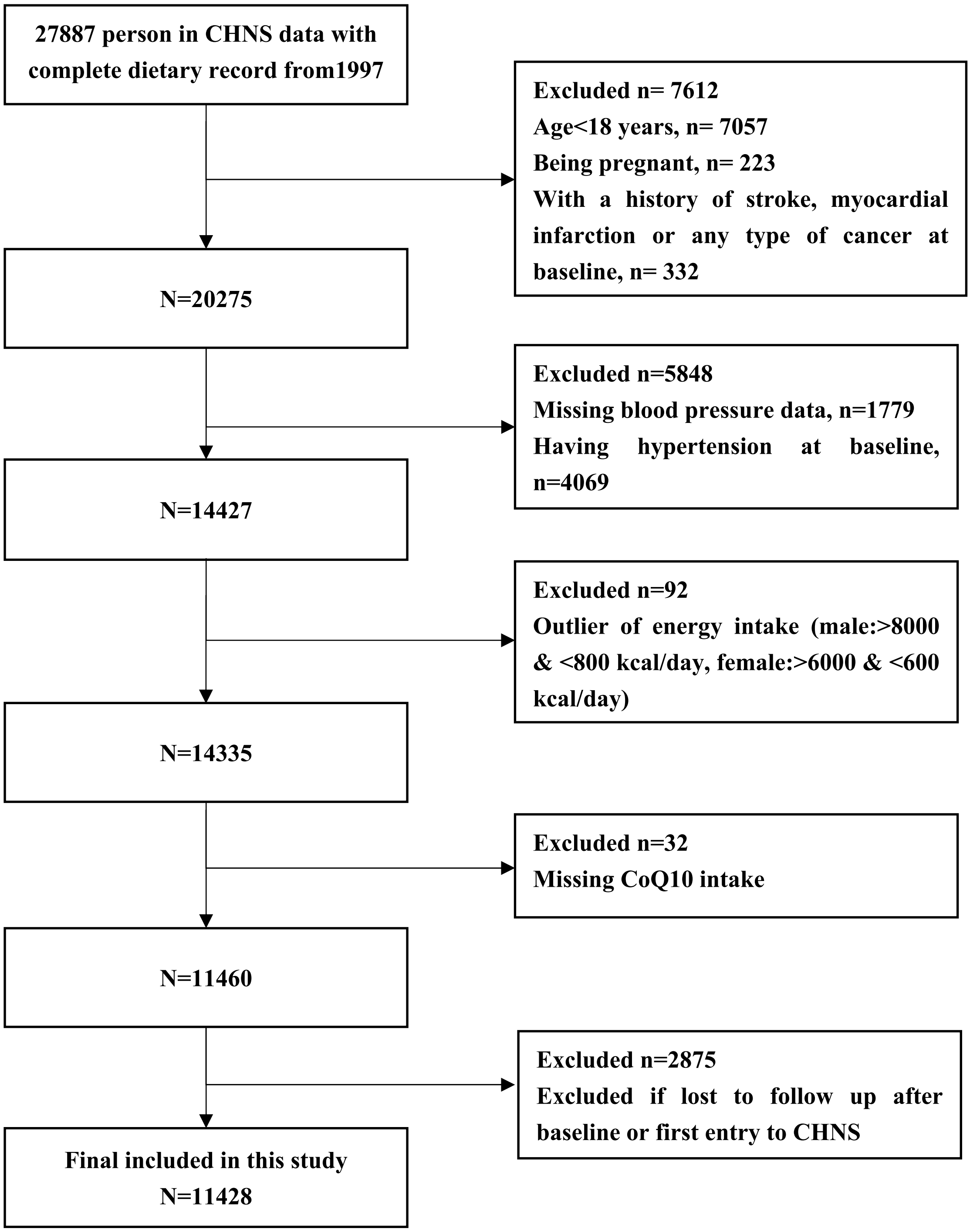

Figure S1. Flow chart

Figure S2. Dose-response relationship between dietary CoQ10 intake and new-onset hypertension across sensitivity analyses

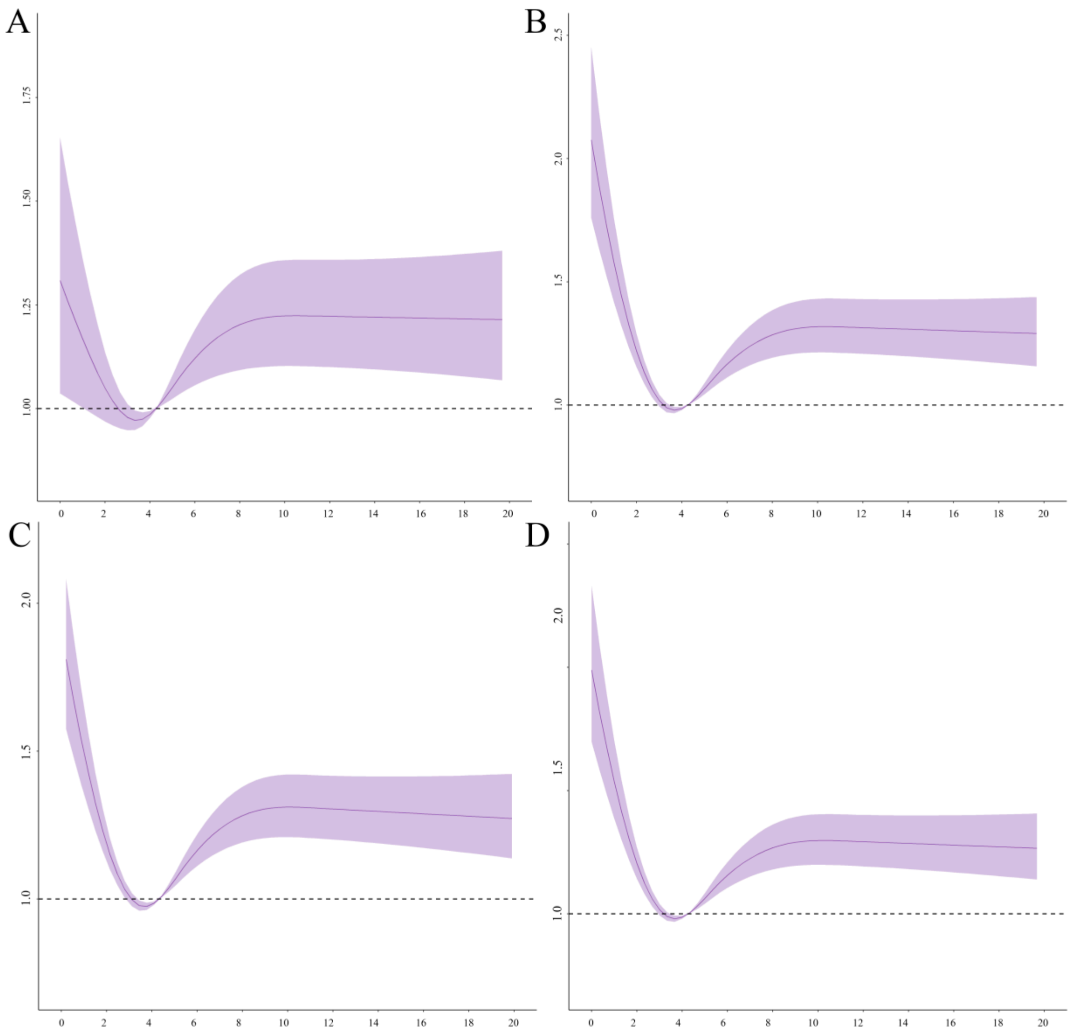

A. After excluding new-onset hypertension occurring during the first 2 years of follow-up; B. The follow-up person-time was calculated as from baseline until the first hypertension diagnosis; C. energy-adjusted residues of dietary CoQ10 intake; D. Use a multiple imputation procedure with 5 rounds of imputation.

Adjusted for age at baseline (continuous), sex (male/female), BMI (continuous), smoking (yes/no) and drinking status (yes/no), education (middle school or below, high school or college or above), residence (urban or rural), marital status (yes/no), physical activity (continuous), as well as cumulative average vegetables (continuous), fruits (continuous), and energy intake (continuous). Solid lines represent point estimates and ribbon represent 95% CIs.

Abbreviations: BMI, Body Mass Index; CI, Confidence Interval.

Table S1. Missing covariates

Covariates	N (%)
Physical activity	330 (2.89)
Education level	226 (1.98)
Alcohol consumer	130 (1.14)
Married status	98 (0.86)
BMI	64 (0.56)
Former or current smoker	51 (0.45)
Energy	4 (0.04)
Total carbohydrate intake	4 (0.04)
Total fat intake	4 (0.04)
Total protein intake	4 (0.04)

Table S2. Participants characteristics by sex *

Characteristics	Total	Male	Female	P
Participants, n	11428	5220	6208	-
CoQ10 intake, mg/d	4.3 [2.7;6.4]	4.5 [2.9;6.7]	4.1 [2.6;6.2]	<0.001
Age, years	41.7 (13.9)	41.8 (14.1)	41.6 (13.8)	0.657
BMI, kg/m^2	22.5 (3.2)	22.4 (3.1)	22.5 (3.3)	0.043
SBP, mmHg	114.1 (11.5)	116.1 (10.6)	112.3 (11.9)	<0.001
DBP, mmHg	74.2 (7.8)	75.5 (7.4)	73.2 (8.0)	<0.001
Married, (n %)	9544 (84.2%)	4209 (81.3%)	5335 (86.7%)	<0.001
Education level, (n %)	3363 (29.6%)	3157 (60.7%)	206 (3.3%)	<0.001
Middle school or below	7912 (70.6%)	3448 (67.1%)	4464 (73.6%)	
High school	2452 (21.9%)	1259 (24.5%)	1193 (19.7%)	
College or above	838 (7.5%)	431 (8.4%)	407 (6.7%)	
Former or current smoker, (n %)	3363 (29.6%)	3157 (60.7%)	206 (3.3%)	<0.001
Alcohol consumer, (n %)	3918 (34.7%)	3248 (62.7%)	670 (11.0%)	<0.001
Urban residence, (n %)	7032 (61.5%)	3239 (62.0%)	3793 (61.1%)	0.307
Physical activity, METs-h/week, median [IQR]	88.9 [18.0;206.4]	104.6 [27.0;234.8]	79.1 [15.4;185.7]	<0.001
Energy, kcal/d	2144.3 (564.0)	2343.2 (573.2)	1977.1 (498.2)	<0.001
Total fat, % of energy	29.8 (10.5)	29.1 (10.4)	30.3 (10.5)	<0.001
Total carbohydrate, % of energy	56.8 (11.3)	56.9 (11.5)	56.7 (11.1)	0.488
Total protein, % of energy	12.7 (2.7)	12.6 (2.7)	12.8 (2.8)	<0.001
Vegetable intake, g/day	88.9 [18.0;206.4]	104.6 [27.0;234.8]	79.1 [15.4;185.7]	<0.001
Fruit intake, g/day	88.9 [18.0;206.4]	104.6 [27.0;234.8]	79.1 [15.4;185.7]	<0.001

[a] Continuous variables are presented as mean (SD), median (IQR) or categorical variables are presented as n (%)

Table S3. Association between dietary CoQ10 intake and new-onset hypertension, excluding cases diagnosed within the first two years of follow-up

CoQ10 intake, g/day	No. of cases (person-years)	Crude model		Adjusted model [a]	
		HR (95% CI)	*P*	HR (95% CI)	*P*
Quartiles					
Q1 (≤ 2.75)	603 (21078)	Ref	-	Ref	-
Q2 (2.75 to ≤ 4.27)	662 (23874)	0.90 (0.81, 1.01)	0.066	0.95 (0.84, 1.06)	0.300
Q3 (4.27 to ≤ 6.35)	664 (23666)	0.92 (0.83, 1.03)	0.150	0.97 (0.86, 1.09)	0.600
Q4 (> 6.35)	649 (21140)	1.08 (0.97, 1.21)	0.200	1.16 (1.03, 1.31)	0.016

[a] Multivariate hazard ratios (95%CIs) adjusted for age at baseline (continuous), sex (male/female), BMI (continuous), smoking (yes/no) and drinking status (yes/no), education (middle school or below, high school or college or above), residence (urban or rural), marital status (yes/no), physical activity (continuous), as well as vegetables (continuous), fruits (continuous), and energy intake (continuous)

Table S4. Association between dietary CoQ10 intake and new-onset hypertension, with follow-up person-time calculated from baseline to initial diagnosis of hypertension

CoQ10 intake, mg/day	No. of cases (person-years)	Crude model		Adjusted model [a]	
		HR (95% CI)	*P*	HR (95% CI)	*P*
Quartiles					
Q1 (≤ 2.71)	1023 (21004)	Ref	-	Ref	-
Q2 (2.71 to ≤ 4.26)	969 (25012)	0.78 (0.71, 0.85)	<0.001	0.82 (0.75, 0.90)	<0.001
Q3 (4.26 to ≤ 6.39)	982 (24568)	0.80 (0.74, 0.88)	<0.001	0.84 (0.77, 0.92)	<0.001
Q4 (> 6.39)	1032 (21504)	0.99 (0.91, 1.08)	0.900	1.02 (0.92, 1.12)	0.700

[a] Multivariate hazard ratios (95%CIs) adjusted for age at baseline (continuous), sex (male/female), BMI (continuous), smoking (yes/no) and drinking status (yes/no), education (middle school or below, high school or college or above), residence (urban or rural), marital status, physical activity (continuous), as well as vegetables (continuous), fruits (continuous), and energy intake (continuous)

Table S5. Association between energy-adjusted residues of dietary CoQ10 intake and new-onset hypertension

CoQ10 intake, mg/day	No. of cases (person-years)	Crude model		Adjusted model [a]	
		HR (95% CI)	*P*	HR (95% CI)	*P*
Quartiles					
Q1 (≤ 2.71)	1085 (22420)	Ref	-	Ref	-
Q2 (2.71 to ≤ 4.26)	965 (24956)	0.80 (0.73, 0.87)	<0.001	0.83 (0.76, 0.91)	<0.001
Q3 (4.26 to ≤ 6.39)	977 (23779)	0.85 (0.78, 0.93)	<0.001	0.89 (0.81, 0.97)	0.010
Q4 (> 6.39)	979 (20932)	0.98 (0.90, 1.06)	0.600	1.02 (0.93, 1.12)	0.600

[a] Multivariate hazard ratios (95% CIs) adjusted for age at baseline (continuous), sex (male/female), BMI (continuous), smoking (yes/no) and drinking status (yes/no), education (middle school or below, high school or college or above), residence (urban or rural), marital status, physical activity (continuous), as well as vegetables (continuous), fruits (continuous), and energy intake (continuous)

Table S6. Association between dietary CoQ10 intake and new-onset hypertension using a multiple imputation procedure with five rounds of imputation

CoQ10 intake, mg/day	No. of cases (person-years)	Crude model		Adjusted model [a]	
		HR (95% CI)	*P*	HR (95% CI)	*P*
Quartiles					
Q1 (≤ 2.71)	1023 (21004)	Ref	-	Ref	-
Q2 (2.71 to ≤ 4.26)	969 (25012)	0.79 (0.72,0.86)	<0.001	0.83 (0.76,0.91)	<0.001
Q3 (4.26 to ≤ 6.39)	982 (24568)	0.82 (0.75,0.89)	<0.001	0.86 (0.78,0.94)	<0.001
Q4 (> 6.39)	1032 (21504)	0.99 (0.91,1.08)	0.834	1.01 (0.92,1.11)	0.790

[a] Multivariate hazard ratios (95% CIs) adjusted for age at baseline (continuous), sex (male/female), BMI (continuous), smoking (yes/no) and drinking status (yes/no), education (middle school or below, high school or college or above), residence (urban or rural), marital status (yes/no), physical activity (continuous), as well as vegetables (continuous), fruits (continuous), and energy intake (continuous)

Table S7. Relationship between dietary CoQ10 intake in three equal groups and risk of new-onset hypertension

CoQ10 intake, mg/day	No. of cases/person-years	Crude model		Adjusted model [a]	
		HR (95% CI)	*P*	HR (95% CI)	*P*
Category					
Group 1 (≤ 3.23)	1357 (29086)	Ref	-	Ref	-
Group 2 (3.23 to ≤ 5.52)	1285 (33491)	0.82 (0.76, 0.88)	<0.001	0.87 (0.80, 0.94)	<0.001
Group 3 (> 5.52)	1364 (29510)	1.00 (0.92, 1.07)	>0.900	1.01 (0.93, 1.09)	>0.900

[a] Adjusted for age at baseline (continuous), sex (male/female), BMI (continuous), smoking (yes/no) and drinking status (yes/no), education (middle school or below, high school or college or above), residence (urban or rural), marital status (yes/no), physical activity (continuous), as well as cumulative average vegetables (continuous), fruits (continuous), and total energy intake (continuous).

Abbreviations: BMI, body mass index; HR, Hazard Ratio; CI, Confidence Interval

Table S8. Relationship between dietary CoQ10 intake in five equal groups and risk of new-onset hypertension.

CoQ10 intake, mg/day	No. of cases/person-years	Crude model		Adjusted model [a]	
		HR (95% CI)	*P*	HR (95% CI)	*P*
Five categories					
Group 1 (≤ 2.36)	837 (16234)	Ref		Ref	
Group 2 (2.36 to ≤ 3.64)	771 (19389)	0.77 (0.69, 0.84)	<0.001	0.80 (0.72, 0.88)	<0.001
Group 3 (3.64 to ≤ 4.96)	767 (20285)	0.73 (0.66, 0.80)	<0.001	0.81 (0.73, 0.89)	<0.001
Group 4 (4.96 to ≤ 7.04)	817 (19314)	0.82 (0.74, 0.90)	<0.001	0.84 (0.76, 0.93)	0.001
Group 5 (> 7.04)	814 (16865)	0.94 (0.85, 1.04)	0.200	0.98 (0.88, 1.09)	0.700

[a] Adjusted for age at baseline (continuous), sex (male/female), BMI (continuous), smoking (yes/no) and drinking status (yes/no), education (middle school or below, high school or college or above), residence (urban or rural), marital status (yes/no), physical activity (continuous), as well as cumulative average vegetables (continuous), fruits (continuous), and total energy intake (continuous). Abbreviations: BMI, body mass index; HR, Hazard Ratio; CI, Confidence Interva
